# Insight into the Influence of Lactic Acid Bacteria Fermentation on the Variations in Flavor of Chickpea Milk

**DOI:** 10.3390/foods11162445

**Published:** 2022-08-13

**Authors:** Xue Zhang, Wenli Tian, Bijun Xie, Zhida Sun

**Affiliations:** 1 College of Food Science and Technology, Huazhong Agricultural University, Wuhan 430070, China; 2 College of Food Engineering, Henan University of Animal Husbandry and Economy, Zhengzhou 450011, China; 3 Institute of Apicultural Research, Chinese Academy of Agricultural Sciences, Beijing 100093, China

**Keywords:** chickpea milk, plant-based, yam addition, GC-MS, electronic nose, odorants substances

## Abstract

This study aimed to evaluate the influence of fermentation on the levels of free amino acids (FAAs) and variations of volatile odorants in four groups of chickpea milk. Electronic nose (E-nose) and gas chromatography–mass spectrometry (GC-MS) data were subjected to mutual validation. W2S and W3S sensors of E-nose were sensitive to volatile constituents in the four groups of unfermented and fermented specimens. After fermentation, the levels of FAAs in the four groups of specimens decreased to varying degrees. Additionally, there were remarkable differences in the types and contents of volatile odor substances in all specimens before and after fermentation. The principal component analysis findings based on E-nose identified the changes of volatile odorants in all specimens before and after fermentation. GC-MS identified 35 and 55 volatile flavor substances in unfermented and fermented specimens, respectively. The varieties of volatile odor substances in fermented chickpea milk (FCM) with papain treatment plus yam addition (38) were more than those in FCM (24), indicating that the coupled treatment of enzymolysis and yam addition could enrich the volatile odorants in fermented specimens. After probiotic fermentation, the contents of off-flavor substances decreased to a certain extent, and key aroma substances such as 2,3-pentanedione, 2,3-butanedione, and heptyl formate were detected. These results demonstrated that lactic acid bacterial fermentation on the basis of enzymolysis and yam addition could be utilized as a feasible approach to improve the flavor of plant-based products adopting chickpea as the original ingredient.

## 1. Introduction

Currently, with the promotion of environmental sustainability and health awareness, nutritious plant-based protein beverages, such as legume milk, have attracted the increasing attention of consumers [1,2]. These plant-based dairy replacements possess a reputation for “health ingredients” such as increased food safety, reduced allergens, an enhanced nutrition profile, and decreased lactose intolerance compared with their dairy counterparts [2,3,4]. Plant-based yogurts produced by the traditional yogurt-making process still have some defects, including unpleasant odor and insufficient phytochemical nutrients. However, they could be modified to improve their acceptability [5]. Hence, the formulas of conventional legume milk products may not be optimal for plant-based systems and should be further explored to improve flavor and quality.

As a substitute to conventional soymilk, chickpea drink is a novel type of nutritious plant-based milk with a high amount of resistant starch and protein, less lipid, and no allergenicity compared with other plant-based beverages [2,6]. With respect to chickpea milk, some research on quality has been conducted into its physicochemical properties, such as viscosity, color, pH, total polysaccharides, etc., [7]. Previous studies found that chickpea drink has a lower appearance rating than its soy counterpart [2,6]. Fermented chickpea milk is a type of yogurt-like drink produced by chickpea milk via lactic acid bacterial fermentation [2]. Lactic acid fermentation may be adopted as an approach to reduce the beany odors in legume foods [8]. It also produces a special fragrance of fermentation, which gives the product a novel and unique flavor. Fermented legume milks possess improved textures and scents and also have certain health-promoting benefits, containing probiotics, high bioactivity, absence of antinutrients, and improved digestibility [9,10,11]. Although fermented legume milk is beneficial to the health of consumers, it has not yet been widely accepted because of its off-flavors [12]. The flavor of food is an important indicator of the quality of products. The main ingredients that affect the flavor of legume milk include undesirable beany flavor substances, e.g., hexanal, 1-ocene-3-alcohol, 1-hexanol, trans-2-ocene aldehyde, and pelargonic aldehyde [13,14,15]. Methods for decreasing the unpleasant-flavor-related odorants in legume milk, such as modified pulse with loss of lip-oxygenase (LOX) genes and improved breeding technology, have been used to prepare legume milk with decreased off-flavor [12,13,16]. Few studies have used electronic nose (E-nose) and gas chromatography–mass spectrometry (GC-MS) to investigate the aroma characteristics of different formulas of chickpea milks before and after fermentation. Limited data are available regarding the influence of nutrient substance addition and enzymatic treatment on the volatile odor constituents of chickpea milk and the flavor variation before and after fermentation. Accordingly, it is necessary to discuss the effect of using probiotic fermentation and explore the feasibility of enzymolysis and yam addition on the volatile odor constituents of chickpea milk.

Chinese yam is a well-known homology of medicine and food and is favored by consumers for its soft and delicate taste [7]. Previous studies reported that the addition of nutrient substances could improve the flavor of legume milk [17,18,19]. However, the effect of yam addition on the flavor of legume milk has not yet been reported. A number of investigations reported that enzymolysis affects the volatile components and corresponding flavor of plant-based systems. For example, aroma substances were released after enzymolysis. Meanwhile, undesirable odorants were reduced due to the liberation of conjugate glycoside bonds of odorants [2,20,21]. As an odor modification enzyme, papain plays a crucial role in increasing the quality of legume milk [2]. Hence, the off-flavors in chickpea milk might be alleviated, and phytochemical nutritional ingredients might be imparted via the coupled treatment of enzymolysis and yam addition. In this study, four groups of chickpea milk with different treatments were prepared to further investigate the changes in volatile odorants and free amino acids (FAAs) before and after fermentation. The aroma characteristics of chickpea milk with different formulas before and after fermentation were explored by means of E-nose, headspace solid-phase microextraction (HS-SPME), and GC-MS. The changes in the flavor types and contents of chickpea milks before and after fermentation, critical individual aroma ingredients, and different classes of chemical constituents related to different formulas were analyzed so as to provide a possible approach to enhance the nutrition and flavor of plant-based substitutes.

## 2. Materials and Methods

### 2.1. Raw Materials and Chemicals

Untoasted chickpeas (Desi) and yam were obtained from a local supermarket. Papain (10^4^ U/g) were gained from Pang Bo Co. (Nanning, China). All the reagents utilized in this report were analytically pure and acquired from Solabio Co. (Beijing, China).

### 2.2. Preparation of Chickpeas Yam Milk

#### 2.2.1. Flow Chart of the Preparation of Yam Milk and Chickpea Milk

Yam slices → pulverized into powder → grind with 2:7 (*w*/*v*) water → heated at 100 °C (2 min) → inactivated yam milk.

Chickpea → dip in 1:2 (*w*/*v*) water overnight → grind with 1:9 (*w*/*v*) water → sterilized at 100 °C (12 min) → chickpea milk acquired.

#### 2.2.2. Operating Points of Chickpeas Yam Milk

**Figure 1:**
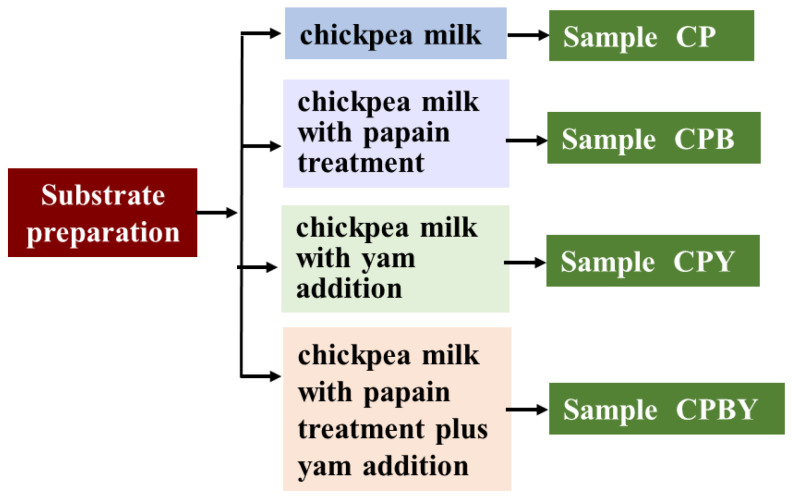
Substrate preparation.

Chickpea yam milks were prepared based on our laboratory literature [7]. Chickpea milk was averagely split into four groups. The first group contained water and chickpea milk at a proportion of 3:8 with non-enzymolysis and marked as CP. The second group was prepared by blending water and chickpea milk at a proportion of 3:8 by adding 80 U papain every chickpea protein gram and was marked as CPB. This group remained at 50 °C (30 min) and was then heated to 100 °C to inactivate the enzyme. The third group was prepared by blending yam milk and chickpea milk at a proportion of 3:8 with non-enzymolysis and was denoted as CPY. The fourth group was prepared by blending yam milk and chickpea milk at a proportion of 3:8 by appending 80 U papain of each chickpea protein gram and was denoted as CPBY. This group remained at 50 °C (30 min) and was then boiled to develop the inactive enzyme. All specimens were appended with sucrose at a proportion of 5% (*v*/*v*) and then stored at 4 °C. The process was shown in Figure 1.

### 2.3. Strains and Fermentation Methods

According to our laboratory literature [7], *lacticaseibacillus rhamnosus* (CICC 20257) was bought from the China Industrial Culture Collection Center (Beijing, China). The foster conditions and methods were based on our previous report [2]. The organism was proliferated (1%, *v*/*v*) twice in fluid medium of MRS [22] and fostered at 37 °C (24 h), unstirring in microaerophilic circumstances.

Four hundred milliliters of chickpea yam milk specimens were acquired vaccinated with 2% (*v*/*v*) (more than 1 × 10^8^ cfu/mL) of an active foster of *lacticaseibacillus rhamnosus* (CICC 20257), and the mixture was cultured at 37 °C (12 h). The specimen was aseptically gathered at 0 and 12 h, instantly refrigerated on ice, and stored at 4 °C until evaluation.

### 2.4. Index Assay for Chickpea Yam Milk Specimens

#### 2.4.1. Assay of FAAs

According to the literature of Yang (2012) and our laboratory research [2], a quantification assay was carried out on an automatic system (A300 AMINO ACID ANALYZER membraPure GmbH.de, Bodenheim, Rhine Gyain, Germany). Compared with known standard substances, a quantitative assay of FAAs was acquired.

#### 2.4.2. E-Nose Identification

The E-nose can be utilized to identify aroma profiles by analyzing specimens and comparing them to known patterns [23]. The sensor reactions of the odor profiles of specimens were determined as per the method of Wu et al., 2016 [23], Jia et al. (2019) [24], and Zhu et al. (2017) [25], with some modifications. The PEN3 nose (Airsense Co., Schwerin, Germany) was connected to 10 metal oxide sensors (W1C, W5S, W3C, W6S, W5C, W1S, W1W, W2S, W2W, W3S) equipped with a headspace sampler, and was utilized for identification. The processes were as follows: 5 g of specimen were placed in a 15 mL headspace vial. The samples were placed in a 50 °C water bath for 30 min. Next, the specimens were removed from the headspace vial, breathed into the chambers, and flushed over the sensors at a rate of 300 mL/min. The specimens were evaluated in three replications.

#### 2.4.3. Pretreatment of Specimens, HS-SPME, and GC-MS 

Eight groups of samples fermented for 0 and 12 h were placed in a No. 90 dish, covered with plastic, freeze-dried (Flexi-Dry TM MP, FTSSYSTEMS Inc., Shenzhen, China), and stored at −20 °C for further determination.

In agreement with the modified method [23,26] and our laboratory literature [2], an HS-SPME assembly bonded with a GC-MS system (7890A, Agilent, San Mateo, CA, USA) was utilized for determining the volatile substances of the specimens. A 5 g sample and 3 mL sodium chloride solution were placed into a 20 mL headspace vial and uniformized with a glass rod for 2 min. The vials and their materials were thermal pretreatments at 60 °C for 20 min to the insertion of the SPME fiber (DVB/CAR/PDMS-50/30 μm, Supe1co, Bellefonte, USA) into the headspace maintained for 40 min. The volatile substances were separated utilizing a DB-WAX chromatographic column (30 m × 0.25 mm × 0.25 μm, Agilent, San Mateo, CA, USA) in the GC-MS system.

The adsorptive SPME fiber was placed into the insertion port of the system after distilling and desorbed at 240 °C (10 min). Helium was utilized as a carrier gas with a mode of unsplit injection. The temperature in the GC column was as follows: initial 50 °C for 3 min, 50 °C to 160 °C at a rate of 3 °C/min, and 160 °C to 250 °C and kept for 10 min. Electron impact was adopted at 70 eV, with a scan range from 33 amu to 350 amu. The ion source temperature and interface temperature were 200 °C and 250 °C, respectively. The mass spectrum of volatiles identified from specimens was compared and matched with the mass spectrometry from NIST 11.0 and the standard substance retention time. Peak area normalization analysis was adopted to obtain the relative content of volatile substances by utilizing an internal standard (2-methyl-3-heptanone). The volatile substances obtained were verified for the match factor greater than 800.

### 2.5. Statistical Analysis 

All tests were conducted with two repetitions, and the data were expressed as means. Duncan’s test assay and principal component analysis were performed using Origin 2021.

## 3. Results and Discussion

### 3.1. Amino Acid Assay of Unfermented and Fermented Specimens

The variations of FAAs in unfermented and fermented specimens are illustrated in Table 1. Overall, the contents of FAAs were reduced in fermented specimens CP-12 h [18.26 mg/kg], CPB-12 h [20.79 mg/kg], CPY-12 h [38.69 mg/kg], and CPBY-12 h [44.51 mg/kg] compared with unfermented specimens CP-0 h [43.02 mg/kg], CPB-0 h [48.05 mg/kg], CPY-0 h [68.51 mg/kg], and CPBY-0 h [79.17 mg/kg]. The content of FAAs in CP yogurt was lower than that in CPY and CPBY, indicating that more FAAs were acquired in yogurts via enzymatic hydrolysis and yam addition. Some FAAs such as Arg, Glu, Ala, and Tyr were remarkably reduced during fermentation. Previous literature showed that lactic acid bacteria possess the ability to synthesize γ-amino butyric acid (GABA) in fermented nutrition [27]. For example, glutamic acid (Glu) can be transformed into GABA via the metabolism of lactic acid bacteria, which can decrease hypertension [28,29]. Furthermore, His and Tyr can be changed into volatile odorant substances, such as benzaldehyde and methyl sulfide [27,30], both of which are helpful for enhancing the aroma of FCM. This is in alignment with the report of Yang (2012). The metabolization of fragrant amino acids, including phenylalanine (Phe) and tyrosine (Tyr), can produce volatile aromatic substances [21,31] during fermentation, suggesting that the utilization of lactic acid bacteria for fermentation could improve the flavor properties to legume-based products [10]. In addition, typical dairy flavor substances (e.g., dimethyl sulfide) can be produced through the degradation of branched-chain amino acids such as Leu, Ile, and Val [32]. The degradation of aromatic amino acids such as Tyr and Phe can form 2-methoxyphenol (guaiacol) with a smoky aroma [32,33]. The above information validated the possible causes of the reduction of FAAs in the four groups of specimens after fermentation.

### 3.2. Response Profile of E-Nose for Unfermented Specimens

#### 3.2.1. Radar Plot of Unfermented Specimens

**Figure 2:**
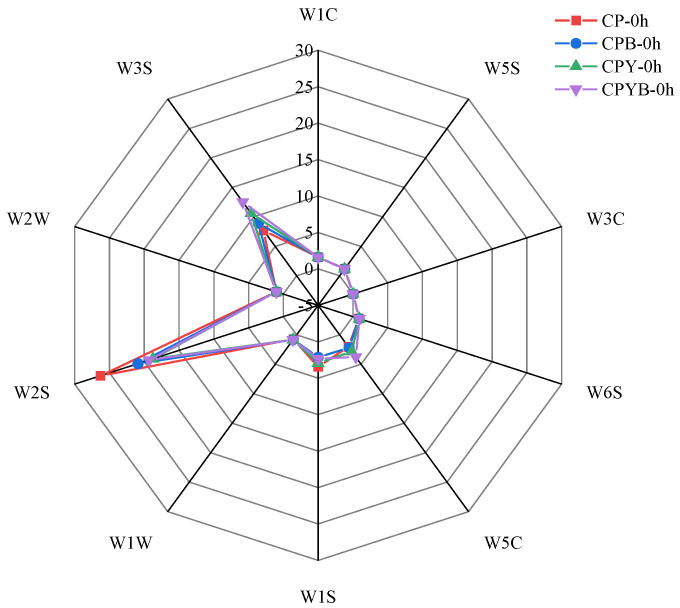
Radar plot of four groups of unfermented specimens. CP-0 h, unfermented chickpea milk; CPB-0 h, unfermented chickpea milk with papain treatment; CPY-0 h, unfermented chickpea milk with yam addition; CPBY-0 h, unfermented chickpea milk with papain treatment plus yam addition.

E-nose makes remarkable contributions to the mode recognition analyses of odors, providing a non-destructive, comprehensive, and quick replacement to evaluate food quality [26,34]. In this study, 10 sensors were utilized for the aroma assay to evaluate the response of volatile substances in four groups of unfermented specimens. The radar chart manifested the response values of four groups of unfermented specimens on the sensory array of the E-nose. The response value of the four groups of unfermented specimens on 10 sensors is shown in Figure 2. The E-nose exhibited fine ability in identifying four groups of unfermented specimens mainly via W2S and W3S sensors (Figure 2). W2S (sensitive to alcohol and aldehyde substances) and W3S (sensitive to alkane substances) sensors bestowed stronger and diverse responses to odorant substances of the four groups of unfermented specimens, suggesting that the unfermented specimens may possess higher abundances of alcohol, aldehyde, ketone, and aliphatic substances. Simultaneously, the W3C (sensitive to ammonia substances) sensor exhibited lower signal strength in the specimens, indicating that it was insensitive to the variations in aroma substances of unfermented specimens. The response values of specimen CPBY-0 h in W5C (sensitive to phenyl substances) and W3S sensors were stronger than those of the other three specimens, indicating that CPBY-0 h may possess higher abundances of alkanes and aliphatic substances. The response values of specimen CP-0 h in W1S (sensitive to methane substances) and W2S sensors were stronger than those of the other three specimens, suggesting that CP-0 h may possess higher abundances of alcohols and aldehydes. The other five sensors were less sensitive to the four groups of unfermented specimens, and the response values were closer, indicating that the volatile substances identified were similar and the signal intensities showed no obvious difference among unfermented specimens. It was difficult to distinguish the four groups of unfermented specimens by merely observing the sensor signals. Thus, principal component analysis (PCA) was conducted in this research.

#### 3.2.2. PCA of E-Nose Dataset of Unfermented Specimens

**Figure 3:**
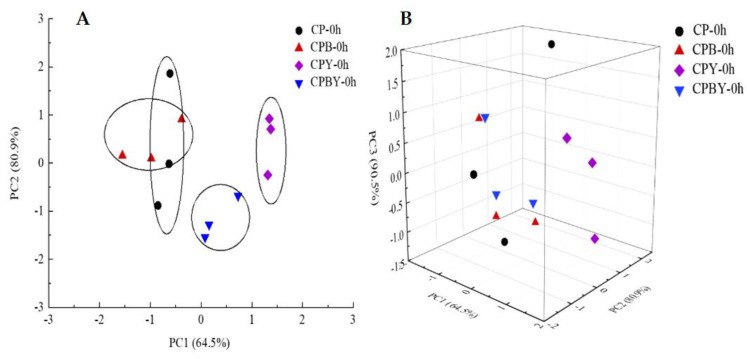
PCA scatter plots (2D and 3D) of four groups of unfermented specimens. (**A**): two-dimension; (**B**): three-dimens ion. CP-0 h, unfermented chickpea milk; CPB-0 h, unfermented chickpea milk with papain treatment; CPY-0 h, unfermented chickpea milk with yam addition; CPBY-0 h, unfermented chickpea milk with papain treatment plus yam addition.

PCA is a linear categorization of the essential information after dimensionality reduction of the E-nose dataset [2,35]. The PCA loading map of four groups of unfermented specimens is shown in Figure 3. As shown in the figure, three PCs were answerable for 64.5% (PC1), 80.9% (PC2), and 90.5% (PC3) of the total contribution rates. The total difference in contribution was higher than 80%, fundamentally overlaying the overarching features of the specimens. The findings revealed that the four groups of unfermented specimens possessed different volatile substances, whereas the distribution in space kept certain regularity. The variations among specimens without the addition of yam chickpea milks (CP, CPB) and with the addition of yam chickpea milks (CPY, CPBY) can be portrayed in PC1. The samples CP-0 h and CPB-0 h were located in the negative areas of PC1 and moved along the negative axis. Both location points were superimposed and near to each other, revealing resemblances in volatile aroma substances. The location of specimens without the addition of yam milk was far from those of samples with the addition of yam milk. The sample CPY-0 h was located in the positive area of PC1 and moved from the positive to the negative area of PC2. The sample CPYB-0 h was distributed in the positive area of PC1 and the negative portion of PC2. Moreover, CPY-0 h and CPYB-0 h were located in the positive areas of PC1, implying that the aroma of samples was dissimilar from those of samples without yam addition and enzymolysis. The above information implied that E-nose could differentiate the flavor of chickpea milk with enzymolysis and yam addition to some degree.

### 3.3. Response Profile of E-Nose for Fermented Specimens

#### 3.3.1. Radar Plot of Fermented Specimens

**Figure 4:**
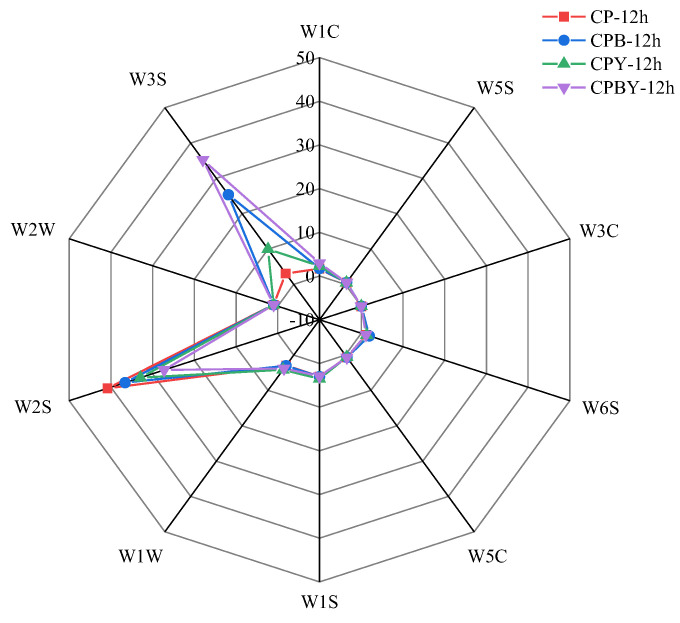
Radar plot of four groups of fermented specimens. CP-0 h, unfermented chickpea milk; CPB-0 h, unfermented chickpea milk with papain treatment; CPY-0 h, unfermented chickpea milk with yam addition; CPBY-0 h, unfermented chickpea milk with papain treatment plus yam addition.

The radar chart of four groups of fermented specimens on 10 sensors is shown in Figure 4. W2S and W3S sensors conferred stronger responses to odorant substances of the four groups of fermented specimens, suggesting that they were sensitive to the variations in aroma substances of fermented specimens. At the same time, the flavor intensity of fermented specimens changed with different formulations, indicating that there were variations in sensor-sensitive substances such as alcohols, aldehydes, ketones, and aliphatic substances. The changes in these ingredients affect the overall flavor of fermented specimens. The response value of specimen CP-12 h in the W2S sensor was remarkably stronger than that of the other three specimens, suggesting that CP-12 h may possess higher abundances of alcohols and aldehydes. The response value of CPBY-12 h in the W3S sensor was stronger than that of the other three specimens, indicating that CPBY-12 h may possess higher abundances of aliphatic substances. Similar to unfermented specimens, the W3C sensor exhibited lower signal intensities than the fermented specimens. The other seven sensors were less sensitive to the four groups of fermented specimens, and the response values were overlapping, indicating that the volatile substances identified were alike, and the signal intensities showed no obvious difference among the fermented specimens.

#### 3.3.2. PCA of E-Nose Dataset of Fermented Specimens

**Figure 5:**
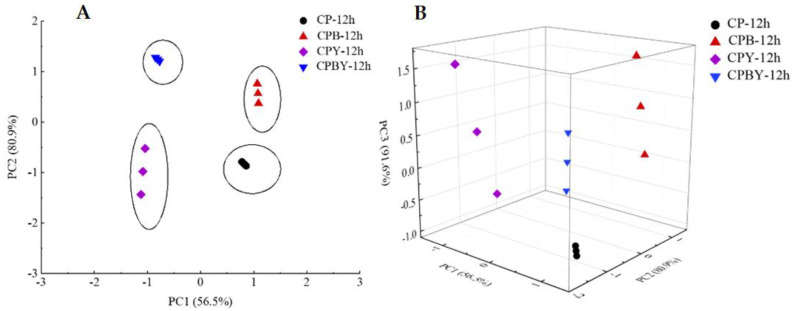
PCA scatter plots (2D and 3D) of four groups of fermented specimens. (**A**): two-dimension; (**B**): three-dimension. CP-12 h, fermented chickpea milk; CPB-12 h, fermented chickpea milk with papain treatment; CPY-12 h, fermented chickpea milk with yam addition; CPBY-12 h, fermented chickpea milk with papain treatment plus yam addition.

Figure 5 show that PC1, PC2, and PC3 of fermented specimens accounted for 56.5%, 80.9%, and 91.6% of the accumulative variability. The total contribution rate was >90%, indicating that the three PCs could explain all the properties of the volatile aroma of fermented specimens. From the perspective of PC2, the specimen CPBY-12 h was distributed in the positive portion of the *y*-axis. CP-12 h and CPY-12 h were located in the negative areas of the *y*-axis. Moreover, the specimens differed chiefly in PC1. CP-12 h and CPB-12 h were distributed in the positive areas of PC1. CPY-12 h and CPBY-12 h were located in the negative area of PC1. The data locations of the four specimens with different means of preparation were dispersed, implying that they possessed varied aromas, and PCA could completely distinguish the aroma substances of the four groups of specimens. Enzymatic hydrolysis and refermentation after adding yam had sufficient effects on the volatile flavor components of chickpea milk. This finding was consistent with that of a previous study, in which chickpea milk specimens that were prepared via dissimilar means displayed diverse spatial locations in PCA [2]. Hence, E-nose can be used to verify the flavor characteristics of chickpea milk before and after fermentation. Nevertheless, accurately identifying the substances in these specimens is difficult. Thus, GC-MS detection was conducted.

### 3.4. GC-MS Assay of Specimens before and after Fermentation

#### 3.4.1. Total Ion Chromatogram of the Four Groups of Samples before and after Fermentation

The total ion chromatograph chart before and after fermentation is illustrated in Figure S1a–d (Supplementary Material). The results indicate that the compositions of volatile substances in the four samples before and after fermentation were different under different processing conditions. Moreover, the total peak areas of volatile substances detected in the specimens after fermentation were remarkably greater than those before fermentation. It was revealed that even though some shared volatile components were present in the four groups of specimens, their contents exhibited certain variations after lactobacillus fermentation. Similar findings were observed by Shi (2017). The alterations of flavor among specimens before and after fermentation can be viewed on the total ion chromatograph charts.

#### 3.4.2. Volatile Flavor Compounds of Four Groups of Unfermented Specimens

The stable balance of various flavor compounds in chickpea milk endows it with unique flavor characteristics. Previous literature described that at least 60 flavor compounds were identified in chickpea milk [15,36], most of which were aldehydes and alcohols, with a small number of ester acids and phenols [19].

**Figure 6:**
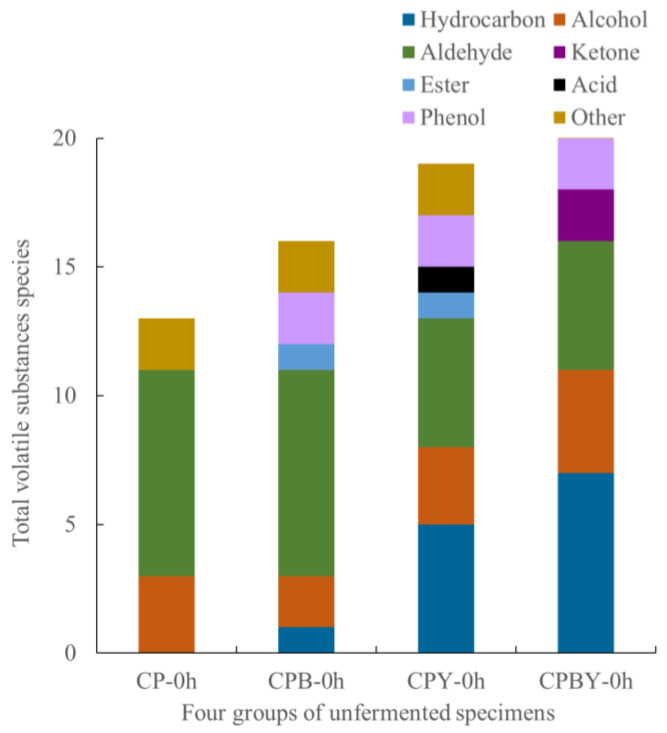
Comparison of volatile odor substances in four groups of unfermented specimens. CP-0 h, unfermented chickpea milk; CPB-0 h, unfermented chickpea milk with papain treatment; CPY-0 h, unfermented chickpea milk with yam addition; CPBY-0 h, unfermented chickpea milk with papain treatment plus yam addition.

As demonstrated in Table 2 and Table 3 and Figure 6, a total of 35 volatile flavor components were identified in the four groups of unfermented specimens, including 6 alcohols, 8 aldehydes, 1 acid, 2 esters, 2 ketones, 12 hydrocarbons, 2 phenols, and 2 other substances. A total of 13, 16, 19, and 22 types of volatile aroma components were detected in CP-0 h, CPB-0 h, CPY-0 h, and CPBY-0 h, respectively. Several vital aroma substances in FCM, including (E)-2-octenal, 1-octene-3-alcohol, hexanol, hexanal, nonanal, and capraldehyde, could be identified in the unfermented specimens. This is in accordance with previous reports, where all of the above odorants were detected in unfermented legume milk [2,37]. Notably, the contents of volatile substances of CPB-0 h, CPY-0 h, and CPBY-0 h unfermented specimens with papain hydrolysis and yam addition were higher than those of CP-0 h without hydrolysis. We hypothesized that enzymatic treatment promoted the oxidative degradation of lipids and proteins, leading to the formation of volatile substances that contribute to the flavor [2].

Previous investigations revealed that there were more than 10 crucial odor-active flavors affecting the aroma of legume milk, including 1-octene-3-alcohol, hexanal, isoamyl alcohol, (E)-2-octenal, and nonanal [2,19]. 1-Octene-3-alcohol and hexanal, which share grassy and agaric-like characteristics, are the most crucial off-flavor substances in legume milk [2,21]. As illustrated in Table 3, hexanal, 1-octene-3-ol, and other flavor components were detected in all four unfermented specimens, which are responsible for the undesirable flavor in chickpea milk. Interestingly, compared with CP specimen (20.91%), the relative amounts of hexanal in CPY, CPB, and CPBY specimens decreased to 15.27%, 8.32%, and 6.58%, respectively. This substance is formed by the conversion of LOX, which catalyzes fatty acids, proteins, and other precursors of legumes and yam [36,38]. The threshold of hexanal was low and had a strong effect on the presentation of flavor. We hypothesized that part of the conjugated hexanal with enzymolysis can be transformed into a free state, accordingly strengthening the degradation of hexanal [2,39]. This finding was in agreement with the result of previous literature [19].

The primary non-beany-flavor ingredients in legume milk mainly include nonanal and (E)-2-octenal [13,19], which present green broccoli, citrus-like, and cucumber flavor [12]. As illustrated in Table 3, nonanal was detected in all four groups of unfermented specimens. Compared with CP specimen (9.79%), the contents of nonanal in CPB, CPY, and CPBY specimens were increased to 13.69%, 11.12%, and 13.06%, respectively. Previous investigations demonstrated that nonanal was degraded by unsaturated fatty acids to form 9-hydroperoxide, which was then decomposed by HPL [19,40]. It was speculated that enzymolysis promoted the degradation of fatty acids, leading to the increase of nonanal. (E)-2-Octenal, with a fatty aroma and strong peanut cake flavor, was obtained from 11-hydroperoxide during the degradation of linolenic acid by LOX [41,42]. Similar to nonanal, compared with those in the CP sample (7.21%), the contents of (E)-2-octenal in CPB and CPBY samples with enzymolysis increased to 8.86% and 8.46%. The contents of heptanal in the CP sample were 7.23%, and that in the CPB sample hydrolyzed by papain was 0.24%. However, the contents of heptanal were not detectable in CP and CPB samples. In general, low aliphatic aldehydes possess unpleasant-smelling odorants. These compounds decrease with the increase of molecular weight. C_8_-C_12_ saturated aldehydes have pleasant aroma characteristics, such as nonanal with rose fragrance and decanal with a refreshing sweet fragrance of flowers. Moreover, certain content of 3-hydroxy-2-butanone (1.52%) and 4-methoxyphenyl ethyl ketone (2.86%) was identified in the CPBY sample, which may endow it with a sweet flavor character. The main component of fresh frankincense is dimethyl sulfide, which has a low threshold and is formed by the degradation of proteins. Dimethyl sulfide was detectable in all four groups of unfermented specimens. Among them, the CPY specimen had the highest content of dimethyl sulfide (1.06%). Some of the flavor compounds in chickpea milk were in a free state, whereas others were in a binding state (conjugated flavor precursors). The conjugated aromatic precursors could be released by enzymatic treatment, resulting in the regeneration, enhancement, and variation of odorants [2]. In addition, compared with the CP specimen, alkane substances and phenol compounds with a slightly fruity aroma were detected in unfermented specimens with yam addition (CPBY and CPY). The above results indicated that the flavor characteristics of the specimens could be improved by papain treatment and yam addition.

#### 3.4.3. Volatile Flavor Substances of the Four Groups of Fermented Specimens

**Figure 7:**
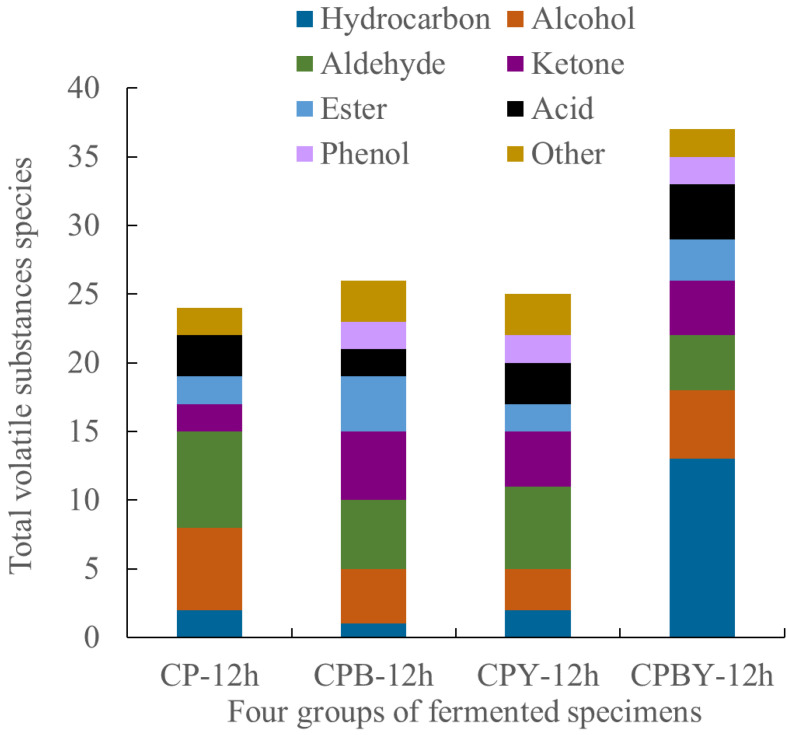
Comparison of volatile odor substances in four groups of fermented specimens. CP-12 h, fermented chickpea milk; CPB-12 h, fermented chickpea milk with papain treatment; CPY-12 h, fermented chickpea milk with yam addition; CPBY-12 h, fermented chickpea milk with papain treatment plus yam addition.

As shown in Table 4 and Table 5 and Figure 7, there were certain differences in the composition and content of odorant compounds in the four groups of fermented specimens. A total of 54 odorant compounds were detected in the four groups of fermented specimens, including 9 alcohols, 7 aldehydes, 5 acids, 6 esters, 7 ketones, 15 hydrocarbons, 2 phenols, and 3 other compounds. A total of 24, 26, 25, and 37 types of volatile aroma components were detected in CP-12 h, CPB-12 h, CPY-12 h, and CPBY-12 h, respectively. Hexanol, 1-octene-3-ol, trans-2-octenal, nonyl aldehyde, capraldehyde, benzaldehyde, acetic acid, caproic acid, 2,3-butanedione, 2,3-pentanedione, formate heptyl, 2-pentanfuran, and dimethyl sulfide were the common odorant compounds in the four group of fermented specimens. After 12 h fermentation of lactic acid bacteria, the types of odorant substances in the four groups of fermented specimens were remarkably increased.

Furthermore, the contents of the main off-flavor substances in the four groups of fermented specimens were reduced to a certain extent. In contrast to unfermented CP (20.91%) and CPY (15.27%), the contents of hexanal in fermented CP and CPY were reduced to 7.29% and 4.58%, respectively, whereas those in fermented CPB and CPBY were not detectable. In a previous study, hexanal in legume milk could be converted into caproic acid through acid dehydrogenase during lactic acid bacterial fermentation [43,44,45]. Consequently, the content of hexanal was reduced or even no longer found in fermented specimens. The above results indicated that the content of hexanal in fermented specimens with enzymolysis was metabolized during fermentation via lactic acid bacteria. Previous studies reported that part of the bound hexanal could be converted into free hexanal after legume milk was enzymatically hydrolyzed, thereby increasing the metabolism of hexanal by lactic acid bacterial proliferation [19,46]. Compared with unfermented CP (16.21%), CPB (14.60%), CPY (11.27%), and CPBY (11.56%), the relative contents of hexanol in fermented specimens decreased to 12.82% (CP), 5.67% (CPB), 9.30% (CPY), and 9.23% (CPBY), respectively. The presence of 1-octene-3-ol, which has a mushroom odor, in fermented specimens has chiefly been linked to 10-hydroperoxide (the precursor substance), which is the degradation product of linoleic acid via the photo-oxidation pathway [19], and also considered to be the main source of the beany odor [46]. Compared with the unfermented specimens, the content of 1-octene-3-ol in fermented CP, CPB, CPY, and CPBY specimens was reduced to 3.42%, 2.93%, 3.17%, and 2.45%, respectively. The heterocyclic compound 2-amyl furan is also a beany component [47]. Compared with unfermented specimens, the contents of this substance in fermented CP, CPB, CPY, and CPBY specimens were reduced to 7.98%, 5.18%, 6.96%, and 4.35%, respectively. The lower content of beany flavor in the fermented specimens obtained in this research compared with unfermented samples might indicate that carbonyl compounds were converted by lactic acid bacterial metabolism into further well-known odor substances, such as ketones, esters, or organic acids (Lee C. 2001), and volatile flavors of chickpea milks were improved.

Moreover, as shown in Table 5, the types and contents of ketones, esters, and organic acids in the four groups of fermented specimens were increased. At the same time, the contents of alcohols and aldehydes were reduced. For example, 2, 3-glutarone and 2, 3-butanedione were not detected in the four groups of unfermented specimens. However, the relative contents of 2,3-glutarone and 2,3-butanedione were 4.88% and 5.36% in CP, 5.81% and 5.66% in CPB, 5.19% and 5.58% in CPY, and 5.39% and 5.71% in CPBY, respectively. These two compounds were similar in flavor and had a creamy and nutty aroma, contributing to the flavor of the cream in milk [48]. In previous literature, these two yogurt-like aroma substances were detected in fermented soymilk [12], resulting in characteristic dairy odors. The occurrence of these two odor substances in FCMs illustrated that they could be formed via citric acid and glucose metabolized by lactic acid bacteria and then under oxidative decarboxylation [49,50]. This was in alignment with the results of a previous study [19].

Ester with fruity aromas [51], such as heptyl formate, was detected in the four groups of fermented specimens with amounts of 2.08% (CP), 3.58% (CPB), 3.25% (CPY), and 2.69% (CPBY), indicating a possible biochemical formation during fermentation. Dihydro-5-amyl-2(3H)-furanone with a strong caramel odor and pleasant peach odor [21] was detected in the CPB specimen with a content of 3.85%, suggesting that the odorant precursor wrapped inside protein aggregates was unfolded by enzymatic hydrolysis and then further formation through amino acid conversion via fermentation occurred [15].

Furthermore, acetic acid and caproic acid could only be identified in the fermented specimens with relative contents of 8.17% and 4.16% in CP, 8.59% and 2.83% in CPB, 5.62% and 2.02% in CPY, and 5.96% and 1.78% in CPBY, respectively. The medium chain fatty acids (C_5_-C_11_) have a greater impact on aroma due to their higher volatility [19]. Caproic acid, with the sweet flavor of cheese and cream, was also an important organic acid substance in yogurt [52]. The above result is in line with that of a previous investigation, revealing a possible formation during lactic acid bacterial proliferation and probably a succeeding diffusion into FCMs [19].

## 4. Conclusions

The findings revealed that the E-nose and GC-MS could efficiently distinguish volatile substances in all unfermented and fermented specimens. The data of E-nose and GC-MS were subjected to mutual validation. The changes in the response value of the four groups of specimens were mainly reflected in W2S and W3S sensors, suggesting that volatile constituents were composed of alcohols, aldehydes, and aliphatic substances. After fermentation, the levels of FAAs in the four groups of specimens were decreased to varying degrees. Additionally, there were remarkable differences in the types and contents of volatile odor substances in all specimens before and after fermentation. The PCA findings based on E-nose can identify the changes of volatile odorants in all specimens before and after fermentation. In total, 35 and 55 volatile flavor substances in unfermented and fermented specimens were identified by GC-MS. CPBY-12 h had more varieties of volatile odor substances (38) than CP-12 h (24), indicating that the coupled treatment of enzymolysis and yam addition could enrich the volatile odorants in fermented specimens and emphasizing the potential of this treatment as a feasible substituent flavor-reforming treatment. After probiotic fermentation, the contents of off-flavor substances were decreased to a certain extent, and key aroma substances such as 2,3-pentanedione, 2,3-butanedione, and heptyl formate were detected. These results demonstrated that lactic acid bacterial fermentation on the basis of enzymolysis and yam addition could be utilized as a feasible approach to improve the flavor of plant-based products adopting chickpea as the original ingredient.

## Figures and Tables

**Table 1:** Variations in the level of free amino acids before and after fermentation.

Samples	CP-0 h	CPB-0 h	CPY-0 h	CPBY-0 h	CP-12 h	CPB-12 h	CPY-12 h	CPBY-12 h
Amino Acid (mg/kg)								
Aspartate (Asp)	0.76	1.19	2.45	2.84	0.09	0.12	1.45	1.39
Threonine (Thr)	0.65	0.80	3.52	4.06	0.50	0.65	0.92	0.96
Serine (Ser)	0.19	0.52	8.03	9.85	0.12	0.18	5.65	6.78
Glutamic acid (Glu)	4.30	5.41	5.60	6.44	1.18	1.29	1.41	1.85
Glycine (Gly)	0.27	0.34	1.66	2.01	0.05	0.16	0.84	1.03
Alanine (Ala)	0.41	0.79	7.63	7.95	0.18	0.22	4.90	5.58
Cysteine (Cys)	0.04	0.07	0.40	0.92	0.03	0.16	0.14	0.12
Valine (Val)	16.23	16.54	16.69	17.35	6.51	6.85	8.16	9.64
Methionine (Met)	0.03	0.07	0.04	0.18	0.02	0.08	0.31	0.27
Isoleucine (Ile)	0.17	0.39	0.51	0.90	0.11	0.17	0.12	0.14
Leucine (Leu)	0.09	0.16	0.22	0.62	0.02	0.03	0.24	0.58
Tyrosine (Tyr)	0.24	0.31	0.15	0.34	0.05	0.12	0.13	0.21
Phenylalanine (Phe)	0.28	0.42	0.45	0.67	0.12	0.29	0.37	0.67
Histidine (His)	0.71	1.06	1.84	1.90	0.36	0.49	1.09	1.32
Lysine (Lys)	2.93	3.14	2.35	4.02	1.16	1.35	0.86	0.85
Arginine (Arg)	15.47	16.01	16.12	17.89	7.53	8.27	11.85	12.19
Proline (Pro)	0.31	0.64	0.98	1.23	0.32	0.46	0.23	0.92
Total	43.08	47.86	68.64	79.17	18.35	20.89	38.67	44.50

Note: Group CP-0 h, unfermented chickpea milk; Group CPB-0 h, unfermented chickpea milk with papain treatment; Group CPY-0 h, unfermented chickpea milk with yam addition; Group CPBY-0 h, unfermented chickpea milk with papain treatment plus yam addition. Group CP-12 h, fermented chickpea milk; Group CPB-12 h, fermented chickpea milk with papain treatment; Group CPY-12 h, fermented chickpea milk with yam addition; Group CPBY-12 h, fermented chickpea milk with papain treatment plus yam addition.

**Table 2:** Volatile odor substances in four groups of unfermented specimens.

Serial Number	Retention Time/Min	Odor Substances	Relative Content (%)			
			CP-0 h	CPB-0 h	CPY-0 h	CPBY-0 h
		**Hydrocarbon**	**0**	**9.81**	**16.49**	**25.49%**
1	3.26	2-propenylidene- cyclobutene	ND	ND	3.09%	ND
2	15.37	1-nonene	ND	9.81%	ND	ND
3	24.95	2-tridecylene	ND	ND	ND	2.00%
4	21.45	1,1,2,3,4,4-hexachloro-1,3-butadiene	ND	ND	3.61%	ND
5	29.35	(Z)-7-hexadecene	ND	ND	2.16%	ND
6	15.84	undecane	ND	ND	ND	3.20%
7	20.69	dodecane	ND	ND	4.80%	9.24%
8	23.67	2-methyl-dodecane	ND	ND	ND	1.48%
9	27.06	6-cyclohexyl-dodecane	ND	ND	ND	1.21%
10	25.36	tridecane	ND	ND	2.83%	ND
11	28.39	3-methyl-tridecane	ND	ND	ND	5.63%
12	24.12	2,4,6-trimethyl-decane	ND	ND	ND	2.73%
		**Alcohol**	**25.32%**	**18.46%**	**19.98%**	**19.03%**
1	7.29	1-hexanol	16.21%	14.52%	11.27%	11.56%
2	10.36	1-Octen-3-ol	4.46%	3.94%	5.67%	3.32%
3	13.01	4-ethyl-1-octyn-3-ol	ND	ND	3.04%	ND
4	14.71	3-decyn-2-ol	4.65%	ND	ND	ND
5	12.06	trans-2-undecen-1-ol	ND	ND	ND	1.69%
6	19.28	1-methyl-4-(1-methylethyl)-cyclohexanol	ND	ND	ND	2.46%
		**Aldehydes**	**64.29%**	**50.95%**	**43.08%**	**39.91%**
1	5.29	hexanal	20.91%	8.32%	15.27%	6.58%
2	8.12	heptanal	7.23%	0.24%	ND	ND
3	12.72	(E)-2-octenal	7.21%	8.86%	7.45%	8.46%
4	13.90	nonanal	9.79%	13.69%	11.12%	13.06%
5	16.03	decanal	5.68%	9.64%	6.65%	9.72%
6	17.58	undecanal	2.55%	2.45%	ND	ND
7	10.12	benzaldehyde	3.36%	3.06%	2.59%	2.09%
8	23.08	pentadecane aldehyde	7.56%	4.69%	ND	ND
		**Ketone**	**0**	**0**	**0**	**4.38%**
1	31.59	2-hydroxy-4-methoxyacetophenone	ND	ND	ND	2.86%
2	25.82	3-hydroxy-2-butanone	ND	ND	ND	1.52%
		**Acids**	**0**	**0**	**3.32**	**0**
1	30.46	2-ethylhexylester-pentanoic acid	ND	ND	3.32	ND
		**Esters**	**0**	**7.43%**	**5.30%**	**0**
1	7.56	hexyl formate	ND	7.43%	ND	ND
2	11.91	3-methyl-oxirane-2-carboxylic acid-methyl ester	ND	ND	5.30%	ND
		**Phenols**	**0**	**4.22%**	**3.62%**	**4.87%**
1	12.16	maltol	ND	3.86%	3.16%	3.95%
2	14.73	2-methoxy-phenol	ND	0.36%	0.46%	0.92%
		**Others**	**10.39%**	**9.13%**	**8.21%**	**6.32%**
1	10.89	2-pentylfuran	9.78%	8.54%	7.15%	5.76%
2	4.05	dimethyl sulfide	0.61%	0.59%	1.06%	0.56%

Note: ND means not detected.

**Table 3:** Types of volatile odor substances in four groups of unfermented specimens.

Sample	CP-0 h	CPB-0 h	CPY-0 h	CPBY-0 h	Sum	Shared
Type						
Hydrocarbon	0	1	5	7	12	0
Alcohol	3	2	3	4	6	2
Aldehydes	8	8	5	5	8	5
Ketone	0	0	0	2	2	0
Esters	0	1	1	0	2	0
Acids	0	0	1	0	1	0
Phenols	0	2	2	2	2	0
Other	2	2	2	2	2	2
Total	13	16	19	22	35	9

**Table 4:** Volatile odor substances in four groups of fermented specimens.

Serial Number	Retention Time/Min	Odor Substances	Relative Content (%)			
			CP-12 h	CPB-12 h	CPY-12 h	CPBY-12 h
		Hydrocarbon	6.18%	3.51%	6.47%	19.82%
1	24.98	(E)-2-tetradecene	ND	ND	ND	1.10%
2	29.35	(Z)-4-tetradecene	ND	ND	ND	1.34%
3	33.12	(E)-3-eicosene	ND	ND	ND	1.51%
4	19.56	caryophyllene	1.83%	ND	ND	ND
5	16.08	undecane	ND	ND	ND	0.37%
6	15.89	dodecane	4.35%	ND	3.26%	3.85%
7	25.16	tridecane	ND	ND	3.21%	3.56%
8	29.68	tetradecane	ND	ND	ND	1.50%
9	28.41	3-methyl-tridecane	ND	ND	ND	1.69%
10	27.10	4-cyclohexyl-tridecane	ND	ND	ND	0.48%
11	36.54	3-methyl-pentadecane	ND	ND	ND	1.00%
12	28.16	10-methyl-eicosane	ND	ND	ND	0.78%
13	35.48	4-cyclohexyl-decane	ND	ND	ND	0.91%
14	6.09	propyl-cyclopropane	ND	3.51%	ND	ND
15	34.01	2,6,10,15-tetramethyl- heptadecane	ND	ND	ND	1.73%
		**Alcohol**	**24.23%**	**16.19%**	**16.87%**	**17.97%**
1	7.29	hexyl alcohol	12.82%	5.67%	9.30%	9.23%
2	6.95	(Z)-3-hexen-1-ol	1.56%	ND	ND	ND
3	10.56	1-octen-3-ol	3.42%	2.93%	3.17%	2.45%
4	30.52	2-hexyl-decanol	ND	ND	ND	0.52%
5	39.61	1-hexadecanol	ND	ND	ND	0.46%
6	13.05	1-octanol	2.13%	ND	ND	ND
7	15.28	1-nonanol	3.09%	ND	ND	ND
8	19.90	nerolidol	1.21%	1.08%	ND	ND
9	14.61	(Z)-2-Octen-1-ol	ND	6.51%	4.40%	5.31%
		**Aldehydes**	**30.83%**	**22.68%**	**25.73%**	**18.61%**
1	5.36	hexanal	7.29%	ND	4.58%	ND
2	8.32	heptanal	4.78%	ND	ND	ND
3	12.70	(E)-2-octenal	2.54%	5.40%	4.35%	5.06%
4	10.13	benzaldehyde	3.02%	2.65%	2.23%	1.90%
5	13.85	nonanal	5.60%	6.68%	5.86%	6.26%
6	16.02	capraldehyde	4.51%	4.60%	5.91%	5.39%
7	21.89	myristic aldehyde	3.09%	3.35%	2.80%	ND
		**Ketone**	**10.24%**	**21.65%**	**15.84%**	**17.81%**
1	18.71	dihydro-5-pentyl-2(3H)-furanone	ND	3.85%	ND	ND
2	31.56	2,2,7-trimethyl-octa-5,6-dien-3-one	ND	ND	ND	4.08%
3	11.82	2(1H)-5-chloro-4,6-diphenyl- pyrimidinone	ND	ND	2.59%	ND
4	10.90	2,3-butanedione	5.36%	5.66%	5.58%	5.71%
5	14.06	2,3-pentanedione	4.88%	5.81%	5.19%	5.39%
6	25.82	3-hydroxy-2-butanone	ND	1.73%	2.48%	2.63%
7	4.51	2-hydroxy-3-pentanone	ND	4.60%	ND	ND
		**Acids**	**15.52%**	**11.42%**	**12.50%**	**10.39%**
1	30.51	acetic acid	8.17%	8.59%	5.62%	5.96%
2	37.13	caproic acid	4.16%	2.83%	2.02%	1.78%
3	30.18	2-ethylhexylester-2-pentanoic acid	ND	ND	4.86%	0.89%
4	11.96	2-2-hexyl-cyclopropaneacetic acid	3.19%	ND	ND	ND
5	37.65	2-tridecyl ester- methoxyacetic acid	ND	ND	ND	1.76%
		**Esters**	**4.20%**	**10.20%**	**5.96%**	**5.25%**
1	7.92	trichloro-acetic acid-methyl ester	ND	ND	ND	0.50%
2	10.26	formic acid, heptyl ester	2.08%	3.58%	3.25%	2.69%
3	24.95	(Z)-9,17-octadecadiena	2.12%	ND	ND	ND
4	32.18	dichloroacetic acid, tridecyl ester	ND	3.42%	ND	ND
5	33.70	methoxyacetic acid, 2-tetradecyl ester	ND	1.93%	ND	ND
6	15.03	butyric acid ethyl ester	ND	1.27%	2.71%	2.06%
		**Phenols**	**0**	**4.51%**	**4.40%**	**5.13%**
1	12.16	maltol	ND	3.95%	3.76%	4.07%
2	14.71	2-methoxy-phenol	ND	0.56%	0.68%	1.06%
		**Others**	**8.80%**	**9.84%**	**12.23%**	**5.02%**
1	11.06	2-pentyl-furan	7.98%	5.18%	6.96%	4.35%
2	10.52	1,6-anhydro-β-D glucopyranose	ND	4.15%	4.09%	ND
3	4.05	dimethyl sulfide	0.82%	0.51%	1.18%	0.67%

**Table 5:** Types of volatile odor substances in four groups of fermented specimens.

Sample	CP-12 h	CPB-12 h	CPY-12 h	CPBY-12 h	Sum	Shared
Type						
Hydrocarbon	2	1	2	13	15	0
Alcohol	6	4	3	5	9	2
Aldehyde	7	5	6	4	7	4
Ketone	2	5	4	4	7	2
Ester	2	4	2	3	6	1
Acid	3	2	3	4	5	2
Phenol	0	2	2	2	2	0
Other	2	3	3	2	3	2
Total	24	26	25	37	54	13

## Data Availability

The data that support the findings of this study are available from the corresponding author upon reasonable request.

## Supplementary Materials

The following supporting information can be downloaded at: https://www.mdpi.com/article/10.3390/foods11162445/s1, Figure S1: Flavor profiles of four groups of specimens before and after fermentation.
