# Supplementary material for: Insight into the Influence of Lactic Acid Bacteria Fermentation on the Variations in Flavor of Chickpea Milk
Source: Foods. 2022 Aug 13;11(16):2445. doi: 10.3390/foods11162445 (PMC9407473; doi:10.3390/foods11162445)
Supplement: Supplementary file 1 [file foods-11-02445-s001.zip › foods-1830322-supplementary.pdf]

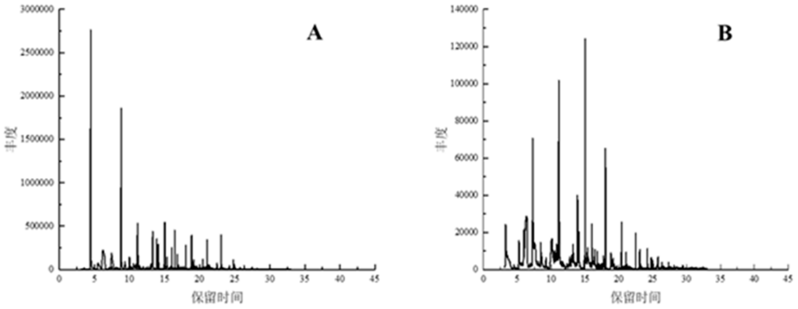

(a)

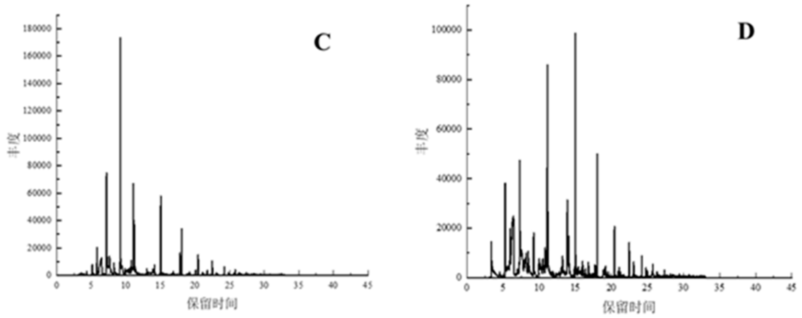

(b)

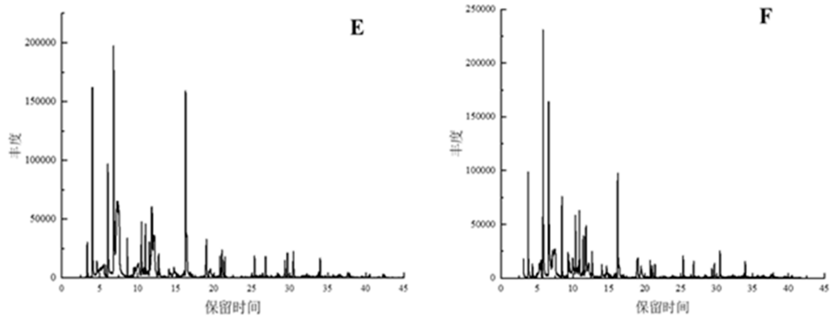

(c)

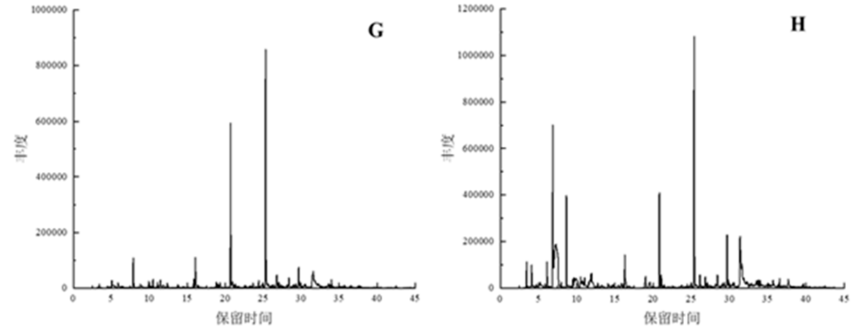

(d)

Supplementary Material Figure S1. Flavor profiles of four groups of specimens before and after fermentation.
Figure S1a. Total ion chromatogram of CP sample before fermentation (A) and after fermentation (B);
Figure S1b. Total ion chromatogram of CPB sample before fermentation (C) and after fermentation (D);
Figure S1c. Total ion chromatogram of CPY sample before fermentation (E) and after fermentation (F);
Figure S1d. Total ion chromatogram of CPBY sample before fermentation (G) and after fermentation (H).
